# The role of synaptic protein NSF in the development and progression of neurological diseases

**DOI:** 10.3389/fnins.2024.1395294

**Published:** 2024-10-21

**Authors:** Jingyue Yang, Lingyue Kong, Li Zou, Yumin Liu

**Affiliations:** ^1^Department of Neurology, Zhongnan Hospital of Wuhan University, Wuhan, China; ^2^Department of Neurosurgery, Zhongnan Hospital of Wuhan University, Wuhan, China

**Keywords:** NSF, SNAREs, synaptic vesicle, neurotransmitter, neurodegeneration

## Abstract

This document provides a comprehensive examination of the pivotal function of the *N*-ethylmaleimide-sensitive factor (NSF) protein in synaptic function. The NSF protein directly participates in critical biological processes, including the cyclic movement of synaptic vesicles (SVs) between exocytosis and endocytosis, the release and transmission of neurotransmitters, and the development of synaptic plasticity through interactions with various proteins, such as SNARE proteins and neurotransmitter receptors. This review also described the multiple functions of NSF in intracellular membrane fusion events and its close associations with several neurological disorders, such as Parkinson’s disease, Alzheimer’s disease, and epilepsy. Subsequent studies should concentrate on determining high-resolution structures of NSF in different domains, identifying its specific alterations in various diseases, and screening small molecule regulators of NSF from multiple perspectives. These research endeavors aim to reveal new therapeutic targets associated with the biological functions of NSF and disease mechanisms.

## 1 Introduction

Within the intricate and enigmatic brain network, neurons and synapses facilitate complex functions such as cognition, learning, and memory. Synapses, as the fundamental units for transmitting information between neurons, are pivotal in relaying neural impulses from one cell to the next. In synaptic function, multiple key players work together to ensure efficient communication and information transmission between neurons. Neurotransmitter receptors, such as *α*-amino-3-hydroxy-5-methyl-4-isoxazolepropionic acid (AMPA) receptors (AMPARs) and *N*-methyl-d-aspartate receptors (NMDARs), receive and transduce chemical signals (Kamalova and Nakagawa, 2021). Synaptic vesicles (SVs) are responsible for the storage and release of neurotransmitters, while the postsynaptic density (PSD) regulates synaptic strength and plasticity (Kennedy, 2018). These components collectively form the complex synaptic structure essential for supporting learning, memory, and other higher-order neural functions.

A critical protein integral to these synaptic processes is the *N*-ethylmaleimide-sensitive factor (NSF). Initially identified for its role in endosomal transport within eukaryotic cells (Block et al., 1988), NSF displays ATPase activity that drives the disassembly of soluble NSF-attachment protein receptor (SNARE) complexes. This activity is vital for intracellular vesicle transport and intercellular substance transfer. Specifically, NSF plays a critical role in the cyclic movement of SVs between exocytosis and endocytosis, which is essential for neurotransmitter release and subsequent endocytosis (Bao et al., 2018). Beyond its primary function in vesicle trafficking, NSF also interacts directly with multiple neurotransmitter receptors, such as AMPARs (Hanley, 2007) and dopamine receptors (Chen and Liu, 2010). These interactions influence receptor transport and expression regulation, thereby impacting synaptic plasticity.

Functional abnormalities in NSF are linked to various neurological diseases, including Parkinson’s disease (PD) (Belluzzi et al., 2016), Alzheimer’s disease (AD) (Hashimoto et al., 2019), and epilepsy (Herold et al., 2018). For example, reduced levels of NSF can impair the autophagy process, which is critical for clearing cellular debris and maintaining cellular health. When autophagy is disrupted, it results in the accumulation of pathological proteins that are characteristic of diseases like Parkinson’s and Alzheimer’s. Additionally, in PD, specific disease-related genes, such as leucine-rich repeat kinase 2 (LRRK2), can influence NSF activity through phosphorylation, contributing to disease pathogenesis.

This review aims to provide a comprehensive overview of the diverse roles of NSF in neuronal physiology, elucidate its mechanisms of action at various stages of synaptic function, and explore its correlations with different neurological diseases. By understanding the multifaceted functions of NSF, we can gain further insights into synaptic function and the pathogenesis of related diseases.

## 2 Structure and function of NSF

### 2.1 Structural characteristics of NSF

In the 1980s, Rothman’s laboratory identified a protein that could revive Golgi transport following the inactivation of the Golgi membrane by *N*-ethylmaleimide (NEM). Owing to its sensitivity to NEM, this protein was designated as the N-ethylmaleimide-sensitive factor, or NSF (Block et al., 1988). NSF possesses ATPase activity and the ability to dissociate the SNARE complex (Tagaya et al., 1993), a capability essential for facilitating membrane fusion events (May et al., 1999; Yu et al., 1999). NSF, which shares homology with the Sec18 protein in *Saccharomyces cerevisiae*, is vital for key biological processes such as membrane fusion, intracellular transport, and vesicle trafficking (Wilson et al., 1989). This homology highlights the conserved importance of these proteins across different species. Previous studies have successfully identified a conditional mutant gene of NSF that may lead to defects in membrane transport under restrictive temperature conditions (May et al., 1999; Yu et al., 1999).

**Figure 1:**
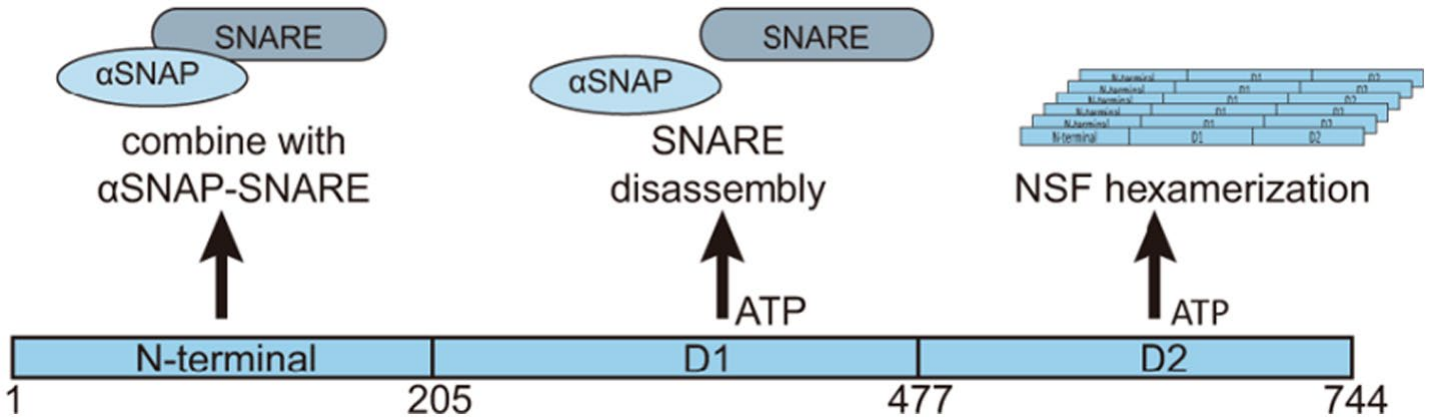
Structure and functions of NSF. NSF is a hexameric protein consisting of six subunits. Each subunit consists of three domains: the N-terminal domain, the D1 domain, and the D2 domain. The N-terminal domain primarily binds to *α*-SNAP and the SNARE complex. The D1 domain has a relatively weak ATP binding capability and is mainly involved in ATP hydrolysis, generating the energy required for dissociating the α-SNAP-SNARE complex. On the other hand, the D2 domain has a higher ATP binding affinity and is responsible for maintaining the hexameric structure of NSF. The different domains of NSF play distinct roles in its ATPase activity and interaction with the SNAP-SNARE complex. Collectively, these domains coordinate various biological functions of NSF.

*N*-ethylmaleimide-sensitive factor is a member of the AAA+ (ATPases Associated with diverse cellular Activities) superfamily. AAA+ ATPases are characterized by a conserved ATPase domain that includes several key regions: the Walker A and B motifs for ATP binding and hydrolysis; the Sensor 1 and Sensor 2 regions for nucleotide sensing; the Second Region of Homology (SRH) essential for oligomerization, and the Nucleotide Binding Domain (NBD), where ATP hydrolysis occurs. The flexible linker within the AAA+ domain allows for the conformational changes required for the enzyme’s mechanical work (Khan et al., 2022). NSF contains a core structure with an ATP binding site region composed of 185 conserved amino acids (Kunau et al., 1993), along with two Type II ATPase loops (Sollner et al., 1993). NSF exists as a homohexamer with a central hole, resulting in a total molecular weight of 500 kDa. Each subunit comprises three domains: the amino-terminal N domain (NSF-N, 1–205), which primarily interacts with *α*-soluble NSF attachment protein (SNAP) and SNARE complexes, a low-affinity ATP-integrated type Walker ATP structure domain (NSF-D1, 206–477), and a second Walker type ATP binding domain (NSF-D2, 478–744) with high ATP affinity (Matveeva et al., 1997; Tagaya et al., 1993; Wilson et al., 1989; Yu et al., 1998). The coordinated activity of these domains allows NSF to execute its cellular functions (Figure 1).

NSF AAA+ is composed of different structural domains with distinct features, each serving different functions. NSF’s D1 domain, part of its AAA+ structure, demonstrates high ATPase activity that is crucial for its overall function (Whiteheart et al., 1994). The 20S supercomplex, a structure formed by NSF, SNAPs, and SNAREs, relies on the D1 domain to facilitate its dissociation (Liu et al., 2013; Nagiec et al., 1995). Upon specific stimulation of SNAP-SNARE, the D1 domain demonstrates ATPase activity, facilitating the dissociation and release of SNARE complexes, thereby playing a crucial role in the membrane fusion process (Matveeva and Whiteheart, 1998; Nagiec et al., 1995). The D1 domain exhibits a relatively weaker ATP-binding activity compared to the D2 domain, as indicated by their dissociation constants (KD) for ATP, which are approximately 15–20 μmol for the D1 domain and 30–40 nmol for the D2 domain (Matveeva et al., 1997). Mutations in the D1 domain, including K266A and E329Q, lead to a substantial reduction in ATPase activity—approximately 70–80% lower than that of the wild type—preventing normal NSF function without SNAP-SNARE complex stimulation (Whiteheart et al., 1994). Additionally, specific mutations, such as R385A, R388A, and D359K, impair NSF’s capacity to assemble into the 20S supercomplex, which is crucial for its function (Nagiec et al., 1995). Arginine fingers within the D1 domain of NSF, specifically Arg385 and Arg388, are essential for its ATPase activity and for the dissociation of the SNAP-SNARE complexes (Zhao et al., 2010).

The D2 domain is widely recognized as the prototype of the AAA+ ATPase domain (Lenzen et al., 1998). It exhibits the highest level of sequence conservation and shares a similar high-level structure with other domains. The D2 domain is composed of two distinct subdomains, known as the *α*/*β* subdomain and the α subdomain, which together are characteristic features of all AAA+ ATPase domains (Lenzen et al., 1998; May et al., 1999; Yu et al., 1998, 1999). The primary function of the D2 domain is to facilitate hexamer formation (Matveeva et al., 1997; Whiteheart et al., 2001). In the NSF hexamer, the K631 residue on one D2 subunit interacts with the *γ*-phosphate of ATP on neighboring D2 subunits within the ring, displacing water molecules that are typically involved in the hydrolysis of the γ-phosphate (King, 2000). The D2 domain comprises a nucleotide-binding subdomain and a C-terminal subdomain with a unique structure among nucleotide-binding proteins, these features are essential for ATP-dependent oligomerization (Yu et al., 1998). The absence of functional arginine fingers in the D2 domain leads to significantly slower ATP hydrolysis (Morgan et al., 1994).

The N-terminal domain of NSF is essential for its binding to the SNAP-SNARE complex, a vital step in cell membrane fusion (Nagiec et al., 1995; Whiteheart et al., 2001; Zhao et al., 2010). The N-terminal domain shares a nearly identical structure with the yeast homolog Sec18 (Babor and Fass, 1999) and the homologous VAT protein from the archaebacterium *Thermoplasma acidophilum* (Coles et al., 1999), indicating structural conservation within the AAA+ ATPase families. The N-terminal domain comprises two subdomains, NA and NB (Lenzen et al., 1998). The domain interface, formed by a groove, may interact with the alpha C-terminal of the SNAP protein (Yu et al., 1999). Tyr83, situated on the loop that connects substructures NA and NB within the N-terminal domain, stabilizes the loop’s structure through hydrogen bonds with adjacent amino acids Gln90 and Lys87 (Zhao et al., 2007). The N domain can recognize various SNAP proteins, which in turn can bind to a range of SNARE complexes (Zhao et al., 2015). Notably, while the N domain only interacts with the C-terminal domain of *α*-SNAP, it does not directly engage with the SNARE complex itself, suggesting a specific selectivity in its molecular interactions (Wimmer et al., 2001). Moreover, NSF can dismantle “dual” SNARE complexes formed by connecting two SNARE complexes head-to-tail (Cipriano et al., 2013). Mutations in the N domain, such as R67, S73, and Q76, impact binding to the SNAP-SNARE complex (Matveeva et al., 2002). The N domain’s structure, while not dispensable for the initial assembly of the NSF hexamer, is crucial for endowing it with functionality; its removal results in the assembly of a non-functional hexameric complex (Whiteheart et al., 1994). The N domain, when isolated, fails to engage with the SNAP-SNARE complex, implying that a multitude of interaction points are indispensable for the effective binding between NSF and its target complex (Nagiec et al., 1995) (Table 1).

**Table 1:** Overview of NSF protein mutations and their impact on functionality and neurological phenotypes.

NSF domain	Mutation	Phenotype	Functional relevance
D1 domain (NSF-D1)	K266A, E329Q	Decreased ATPase activity (70–80% decrease)	Impaires NSF activity in SNARE complex dissociation without SNAP-SNARE stimulation
	R385A, R388A, D359K	Impaired formation of 20S supercomplex	Affects the ability of NSF to form the 20S supercomplex
	Arginine fingers (R385, R388)	ATPase activity and SNAP-SNARE complex dissociation	Crucial for NSF’s role in vesicle fusion
D2 domain (NSF-D2)	R385A, R388A, D359K	Impaired formation of 20S supercomplex	Disruptes SNAP-SNARE complex dissociation, essential for hexamer stability and NSF’s role in vesicle trafficking.
	K631	Hexamer formation disruption	Vital for NSF’s ATPase activity and SNARE complex dissociation
	S569A	Elimination of phosphorylation and stabilization of NSF oligomers	Affects NSF’s oligomerization state and function
	S569E	Oligomerization defects	Affects NSF’s oligomerization state and function
N-Terminal (NSF-N)	R67A, S73A, Q76A	Reduced binding affinity to SNAP-SNARE complex	Impaired vesicle docking and fusion, leading to decreased neurotransmitter release efficiency.
	Tyr83 mutation	Phosphorylation interrupts the interaction of NSF with α-SNAP	Affects the stability of the NSF-SNARE complex and vesicle fusion.
	Cys21/Cys91	*S*-nitrosylation impacts SNARE complex interaction	Modulates synaptic plasticity and neurotransmitter release

The NSF is an ATPase with a highly conserved AAA+ domain structure, existing as a homohexamer with each subunit comprising three domains: an N domain and two domains D1 and D2. These domains have differential ATP-binding capabilities. The N domain is responsible for binding to SNAP and SNARE complexes. The D1 domain has a low affinity for ATP binding and primarily functions in ATP hydrolysis for energy generation. Conversely, the D2 domain displays a high ATP-binding capacity and is crucial for maintaining the hexameric structure of NSF. NSF’s precise recognition and efficient hydrolysis of ATP are instrumental in facilitating the depolymerization of SNARE complexes, thereby exerting a crucial regulatory effect on intracellular membrane transport.

### 2.2 Functional mechanism of NSF

#### 2.2.1 ATPase activity and ATPase activation process

*N*-ethylmaleimide-sensitive factor, a member of the AAA+ ATPase family, can convert chemical energy derived from ATP hydrolysis into mechanical force for various biological tasks, such as DNA unwinding, protein depolymerization, and protein complex dissociation (Hanson and Whiteheart, 2005). The AAA+ ATPase family is widely distributed in prokaryotes and eukaryotes, participating in numerous cellular processes and exhibiting significant functional diversity (Erzberger and Berger, 2006). In 1994, researchers first confirmed that NSF mediates the fusion of vesicles with target membrane bilayers through ATP hydrolysis (Whiteheart et al., 1994). Phylogenetically, NSF belongs to the “classical branch” of the AAA+ protein family (Erzberger and Berger, 2006). Notably, NSF is the sole ATPase capable of restoring SNARE protein activity following membrane-to-membrane fusion (Hong and Lev, 2014). Like other family members, NSF exhibits ATPase activity, with an optimum pH of around 9.0 (Peters et al., 1990). The nucleotide-binding pocket of NSF comprises several conserved motifs, including Walker A, Walker B, Sensor 1, Sensor 2, and the arginine finger motif (Zhao et al., 2012). The lysine residues conserved in the two Walker A motifs (266, 549) are essential for ATP binding. Sensor 1 distinguishes bound ATP from ADP by forming hydrogen bonds with the *γ*-phosphate group of ATP. In NSF-D2 structures, aspartic acid (usually N374) of Sensor 1 is substituted with serine (generally S655), forming a hydrogen bond network that potentially facilitates the ATP hydrolysis reaction (Lenzen et al., 1998).

**Figure 2:**
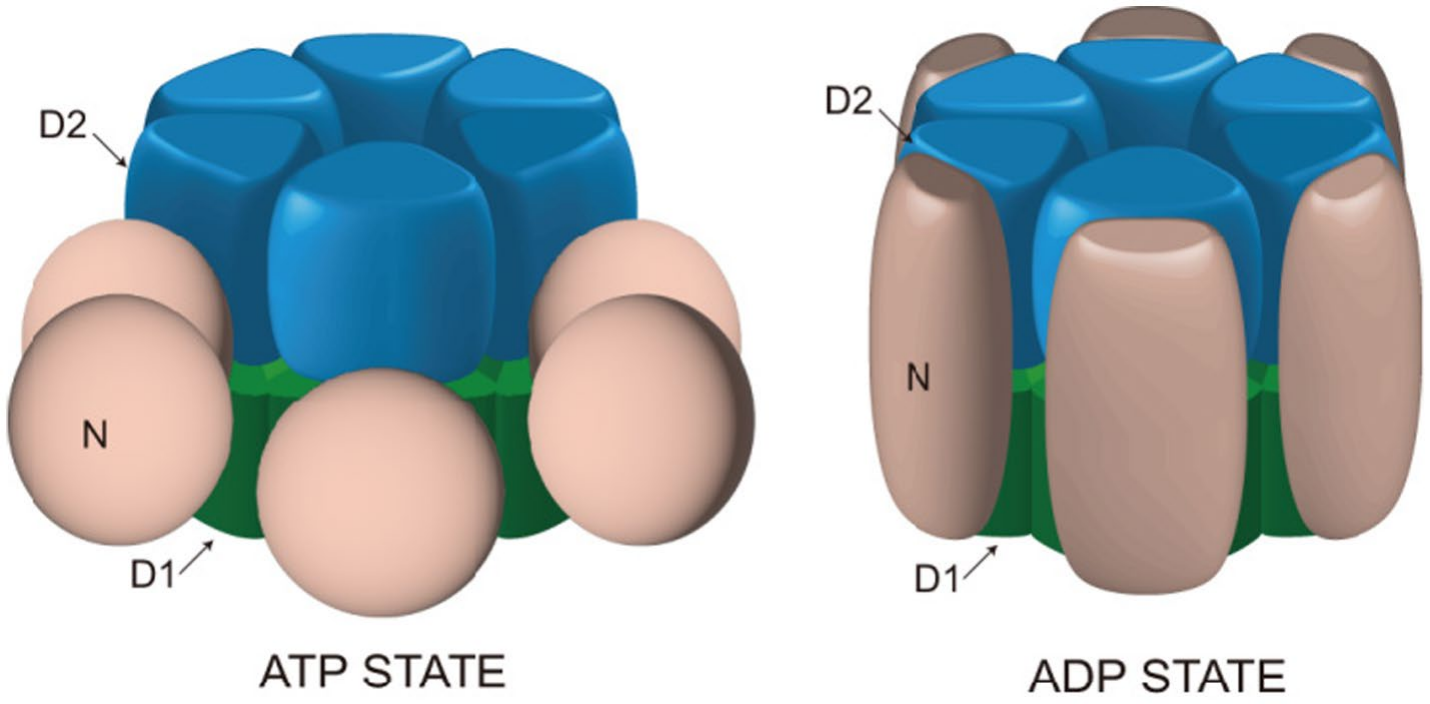
Structural changes of NSF in ATP and ADP binding states. In the presence of ATP, the NSF-D1 domain adopts a double-ring configuration with a diameter of approximately 13 nm, while the NSF-N domain is positioned outside the double ring. Upon binding with ADP, the NSF-D1 domain expands to a diameter of approximately 16 nm, and the NSF-N domain assembles above the double ring, forming a ring-shaped configuration. These structural changes provide the driving force for NSF to facilitate the disassembly of the SNARE complex.

AAA+ proteins generally exhibit the highest affinity for substrates when in the ATP-bound state (Hanson et al., 1997a,b; Tagaya et al., 1993). Freeze-etching electron microscopy studies have revealed different conformations of NSF in the ATP- or ADP-bound state. On mica sheets, NSF forms hexamers with six-fold radial symmetry, NSF-D1 and NSF-D2 domains form a bilayer ring structure. In the ATP-bound state, NSF-D2 remains in its structure, playing an essential role in NSF’s ATPase activity. Meanwhile, NSF-D1 forms a bilayer ring structure, approximately 13 nm in diameter, consisting of six NSF-N domains, located away from the central pore. In the ADP-bound state, the N domains assemble on the double loop structure, resulting in the diameter of the NSF-D1 ring expanding to approximately 16 nm (Hanson et al., 1997a,b) (Figure 2). This conformational transition serves as the driving force for the disassembly of the SNARE complex and represents a crucial process mediated by NSF. Furthermore, NSF interacts with SNAP proteins to regulate its ATPase activity. Peptides that inhibit NSF function also inhibit SNAP-stimulated ATP hydrolysis and transmitter release (Schweizer et al., 1998). Understanding the function and mechanism of NSF is greatly aided by its structural information.

#### 2.2.2 Mechanism of SNARE complex dissociation

The recruitment of NSF to membranes involves adaptor proteins known as soluble NSF attachment proteins (SNAPs), which interact with membrane SNAP receptors (SNAREs) (Wilson et al., 1992). SNAP is a crucial component in the synaptic machinery that interacts directly with NSF to facilitate membrane fusion events. It acts as an adapter protein, bridging NSF and the SNARE complex. Specifically, SNAP binds to SNARE complexes, which are formed by the interaction of synaptobrevin (VAMP), syntaxin, and SNAP-25 (Matveeva and Whiteheart, 1998; Whiteheart et al., 1994; Zhao et al., 2015). Once bound, SNAP recruits NSF to the complex. NSF then utilizes the energy from ATP hydrolysis to disassemble the SNARE complexes, allowing the SNARE proteins to be recycled for subsequent rounds of vesicle fusion and neurotransmitter release (Nagiec et al., 1995; Sollner et al., 1993; Zhao et al., 2010).

**Figure 3:**
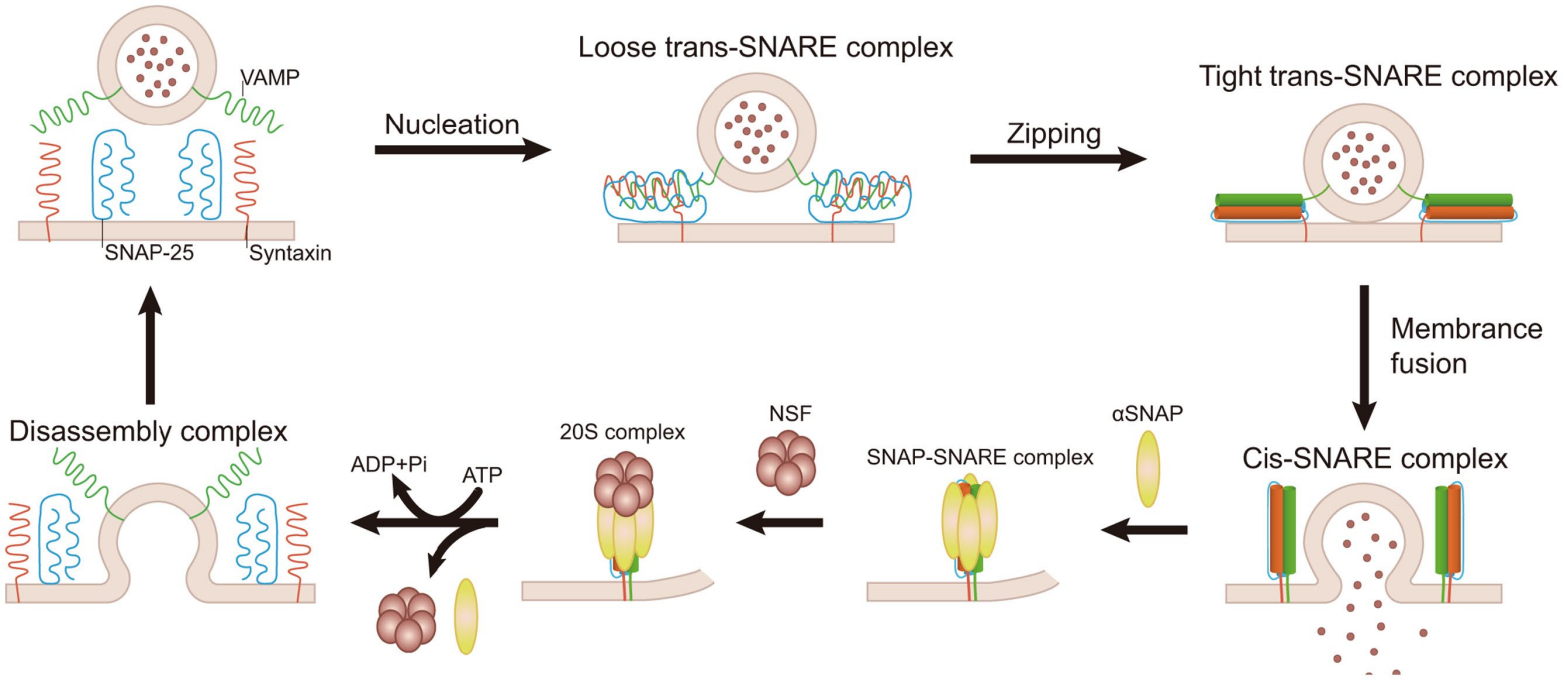
Mechanism of NSF-mediated disassembly of SNARE complex. SNARE proteins initiate the process of a “zipper-like” binding from the N-terminal, which forms a tight link between vesicles and target membranes. It results in the formation of the trans-SNARE complex, which facilitates membrane fusion. Following fusion, a stable cis-SNARE complex is established. NSF interacts with SNAP and, through ATP hydrolysis, dissociates the cis-SNARE complex into individual SNARE proteins. These individual SNARE proteins are subsequently recycled for a new round of membrane fusion.

SNARE proteins are essential for intracellular vesicle transport, such as substance transport through the Golgi apparatus, and secretion processes, such as neurotransmitter release (Malsam and Sollner, 2011; Rothman, 2014). SNARE proteins facilitate the fusion between different membrane tissues by interacting between vesicle SNAREs (v-SNAREs) and target SNAREs (t-SNAREs) (Hong and Lev, 2014). Before membrane fusion, the interaction of v-SNAREs and t-SNAREs brings two cellular membrane organelles nearby, creating conditions for fusion (Sutton et al., 1998; Weber et al., 1998). The SNARE complex, consisting of Synaptobrevin/VAMP, syntaxin-1, and SNAP-25, is arranged parallelly. The assembly process begins at the N-terminal and progresses toward the C-terminal, forming a parallel four-helix structure via a “zipper-like” binding mechanism (Hanson et al., 1997a,b; Pick et al., 2018). Ultimately, v-SNAREs and v-SNAREs bind to each other, forming a helical bundle that links the SV to the cell membrane, and with the coordinated action of the calcium sensor synaptotagmin-1 (syt1), initiating the opening of the fusion pore (Bao et al., 2018). The resulting structure is known as a *trans*-SNARE complex, or more commonly, SNAREpins (Pick et al., 2018). After the fusion of membranes, SNARE proteins form stable complexes at the anchoring site on the same membrane, referred to as cis-SNARE complexes. Following membrane fusion, the inactive SNARE complexes need to be regenerated through the ATPase activity of the NSF in preparation for the next round of membrane fusion (Hong and Lev, 2014). NSF interacts with a variety of SNARE complexes through SNAP adaptors. The hydrophobic N-terminal loop of SNAP can be inserted into the cell membrane, interacting with membrane proteins to accelerate the dissociation of SNARE protein complexes (Winter et al., 2009). Before ATP hydrolysis, NSF, SNAPs, and SNAREs form a 20S supercomplex, the initial state of SNARE complex dissociation (Hanson et al., 1997a,b; Malhotra et al., 1988; Sollner et al., 1993). NSF interacts with SNAP to hydrolyze ATP, which results in the disassembly of the post-fusion cis-SNARE complexes and the dissociation of inactive SNARE complexes into active SNAREs, thereby maintaining the availability of individual SNAREs (Sollner et al., 1993). The 20S supercomplex typically forms near the C-terminal four-helix structure of the SNARE complex. This process involves the interaction of several SNAP molecules with both the membrane and the SNARE proteins, facilitating the assembly of the supercomplex (Winter et al., 2009). In the cytoplasm, the 20S supercomplex may be a transient entity, as the ATPase activity of NSF significantly increases upon binding to SNAP and SNARE complexes (Cipriano et al., 2013). Additionally, NSF plays a role in quality control by promoting the formation of fusogenic SNARE complexes through disassembling syntaxin-SNAP-25 binary complexes and preventing the formation of non-canonical pathway complexes (Figure 3) (Brunger et al., 2018; Ma et al., 2013).

Electrostatic interactions are crucial for the disassembly of SNARE complexes. Research highlights the prominence of electrostatic interactions in the binding interfaces between the NSF N-terminal domain and *α*-SNAP and between α-SNAP and the SNARE complex (Marz et al., 2003). The NSF N-terminal domain uses four positively charged residues (R10, R67, K68, and K104) to interact with two sets of negatively charged residues at the C-terminal of α-SNAP (D217, E249, E252, E253 as one set; D290, E291, E292, D293 as another set). Meanwhile, the positively charged residues (K122, K163, K203, R239) of *α*-SNAP interact with the negatively charged surface on the SNARE complex (Zhao et al., 2015). NSF adopts a processive unfolding mechanism to break down SNARE proteins, consuming dozens of ATP molecules to decompose each SNARE complex (Cipriano et al., 2013). NSF can disassemble a single SNARE complex in one round of ATP turnover without requiring ATP replacement (Ryu et al., 2015). This process involves the 20S supercomplex, which functions like a loaded spring. As ATP hydrolyzes, the dissociation of phosphate ions causes the NSF hexamer to undergo internal tension, leading to a tense state. During the subsequent latent period of several tens of seconds, tension accumulates in the 20S supercomplex. Subsequently, NSF rapidly releases the accumulated tension within 20 milliseconds, leading to the breakdown of SNARE proteins and immediate release (Ryu et al., 2015).

The activation of NSF ATPase is indispensable for efficiently dissociating SNARE complexes. Despite normal levels of ATPase activity, mutants of *α*-SNAP and NSF cannot dissociate SNARE complexes (Barnard et al., 1996; Horsnell et al., 2002). The SNAP/SNARE complex has different effects on the ATPase activity of NSF (Matveeva and Whiteheart, 1998). Neuronal SNAREs enhance the ATPase activity of the nervous system by approximately 26 times (Cipriano et al., 2013), demonstrating NSF’s ability to regulate the activity of different SNARE complexes based on the requirements of cellular membrane fusion. The cytoplasmic-free NSF hexamer ATPase is the only active form capable of dissociating SNARE complexes. In contrast, when NSF is deposited, it becomes inactive (Mohtashami et al., 2001). This highlights the importance of NSF’s activity state for its functional role. The binding of ATP to the NSF hexamer occurs randomly and with negative cooperativity, whereas, in the NSF-SNAP-SNARE complex, it happens synchronously and with positive cooperativity. NSF and the SNARE complex exhibit extraordinary cooperativity, preventing ATP consumption without productive disassembly. The binding of NSF to ATP exhibits randomness. However, ATP hydrolysis during the SNARE disassembly process is fully synchronized, minimizing the consumption of ATP by NSF molecules forming non-20S complexes (Kim et al., 2021).

*N*-ethylmaleimide-sensitive factor and SNAP-SNARE complex interactions occur in a 1:1 ratio, meaning each NSF interacts with one SNAP-SNARE complex (Cipriano et al., 2013). Eukaryotic organisms typically have only one form of NSF ATPase, while Drosophila expresses dNSF-1 and dNSF-2 (Boulianne and Trimble, 1995; Mohtashami et al., 2001; Pallanck et al., 1995). There are three homologs of SNAP proteins in mammals: *α*-SNAP and *γ*-SNAP are present ubiquitously, whereas *β*-SNAP is a brain-specific isoform with region-specific expression (Whiteheart et al., 1993). Despite the presence of over 30 different SNARE complexes in typical eukaryotic cells (Jahn and Scheller, 2006; Zhao et al., 2015), NSF is responsible for retrieving all SNARE complexes, highlighting its importance in cellular membrane fusion processes. Electron microscopy analyses indicate the presence of three α-SNAP molecules in purified 20S complexes. The trimeric α-SNAP can independently bind to NSF in the absence of SNARE complexes, while monomeric α-SNAP requires the presence of SNARE complexes to bind to NSF. On the cell membrane, each NSF hexamer requires three α-SNAPs to facilitate binding to SNARE proteins (Wimmer et al., 2001). The C-terminal domains of α-SNAP and γ-SNAP are highly conserved, particularly the penultimate leucine, which is crucial in binding to NSF and activating its ATPase activity (Bitto et al., 2008). Electron microscopy at low temperatures has unveiled an asymmetry in the interaction between NSF and the 20S supercomplex. In this configuration, the ATPase rings align in the same direction, with the N-terminal domain of NSF and SNAP forming a stabilizing anchor for the SNARE complex. This unique arrangement permits the unfolded SNARE complex to dynamically ‘float’ within the space between the NSF N domain’s opening and the D1 ring (May et al., 1999; Yu et al., 1999). Different studies show variations in the disassembly process of SNARE complexes. For 20S supercomplexes containing neuronal SNARE complexes, there are four α-SNAP molecules surrounding the SNARE complex, while for 20S supercomplexes containing VAMP-7 SNARE complexes, only two α-SNAP molecules bind to the SNARE complex. Further confirmation based on composition-gradient multi-angle light scattering (CG-MALS) experiments reveals a maximum ratio of 4:1 for α-SNAP to neuronal SNARE complexes and a maximum ratio of 2:1 for α-SNAP to VAMP-7 SNARE complexes (Zhao et al., 2015).

#### 2.2.3 NSF in postsynaptic receptor function

**Figure 4:**
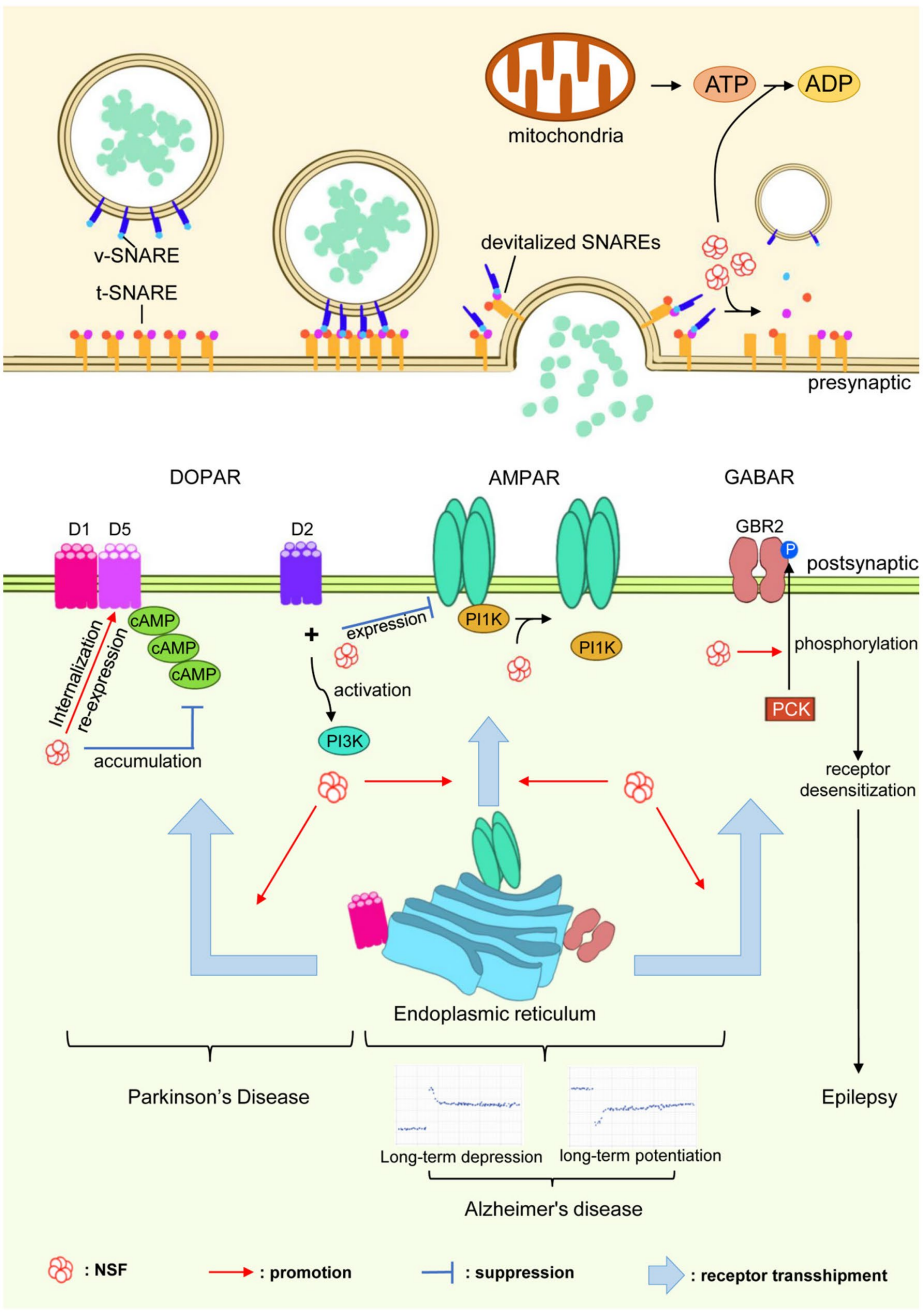
Role of NSF in neurological disorders. NSF, through its interactions with neurotransmitter receptors such as AMPARs, GABARs, and DOPARs, plays a pivotal role in synaptic regulation. This figure demonstrates how NSF influences receptor trafficking, expression, and regulation, which are crucial for synaptic transmission. The depiction underscores NSF’s involvement in the pathogenesis of neurological disorders like Parkinson’s disease, Alzheimer’s disease, and epilepsy, indicating its potential as a key target for therapeutic interventions.

*N*-ethylmaleimide-sensitive factor plays a critical role in the regulation of various postsynaptic receptors, including AMPA receptors (AMPAR), GABA receptors (GABAR), dopamine receptors (DOPAR), gamma-aminobutyric acid receptors (GABARs), β2-adrenergic receptors (β2-AR), β-arrestin1, GATE-16, and Rab, among others. These receptors are essential for synaptic transmission and neuronal communication. By examining how NSF interacts with these receptors, we can better understand its role in influencing their trafficking, expression, and functional regulation. The illustration (Figure 4) shows how NSF is involved in various neurological disorders by regulating and controlling key receptors within synaptic structures.

##### 2.2.3.1 AMPAR

The AMPARs are integral to facilitating rapid excitatory synaptic transmission within the central nervous system (Seeburg, 1993). Composed of four subunits, namely GluA1, GluA2, GluA3, and GluA4, AMPARs can form various subtypes, among which the GluA2 subunit is essential for the formation of mature AMPARs (Gu et al., 2020). The phosphorylation regions located at the C-terminal of AMPARs interact with synaptic proteins, collectively regulating receptor function and their clustering at excitatory synapses (Roche et al., 1994). NSF plays a significant role in governing the function and stability of AMPARs at excitatory synapses (Haas, 1998; Hanley et al., 2002; Osten et al., 1998). Specifically, NSF interacts with the C-terminal domains of the GluR2 and GluR4c subunits of AMPARs, actively participating in the regulation of receptor function and synaptic clustering (Osten et al., 1998; Song et al., 1998). Serving as a disassembly partner, NSF binds to the GluA2 subunit and, using the energy generated from ATP hydrolysis, separates AMPARs from PICK1, thereby inhibiting internalization of GluA2 and stabilizing the presence of the receptor at synapses (Beretta et al., 2005; Song et al., 1998). The interaction between NSF and GluR2 is regulated by SNAP and ATP, similar to the reversible binding of NSF and SNAP to SNARE membrane fusion machinery, thereby shedding light on NSF’s chaperone role in AMPAR processing (Hanley, 2007). The NSF-GluR2 interaction maintains the stability of surface AMPARs on postsynaptic membranes, which is crucial for regulating excitatory synaptic function (Kim and Lisman, 2001). NSF also facilitates the transport and lateral movement of GluA2-containing receptors from extrasynaptic sites to synapses (Beretta et al., 2005), playing a pivotal role in the molecular regulation of AMPARs and intracellular heterotypic membrane fusion events (Whiteheart and Matveeva, 2004).

*N*-ethylmaleimide-sensitive factor is also involved in regulating Calcium-Permeable AMPA Receptor Plasticity (CARP) at synapses. *In vitro* experiments have demonstrated that NSF maintains the number of AMPARs at synapses by disassembling the GluR2-PICK1 complex (Hanley et al., 2002), thus controlling synaptic calcium permeability changes, crucial for CARP modulation. NSF’s role in CARP is especially important in synapses lacking GluR2 and containing GluR2 receptors, where dynamic exchange processes occur (Gardner et al., 2005). Additionally, the binding of NSF to GluR2 is regulated by the calcium concentration, with high levels of calcium inhibiting this interaction and affecting AMPAR transport (Hanley, 2007). Furthermore, the selective interaction between GluR2 and NSF is necessary for maintaining synaptic AMPAR binding and their involvement in long-term depression (LTD) (Steinberg et al., 2004) and late long-term potentiation (LTP) (Yao et al., 2008). The NSF/GluR2 interaction plays a crucial role in both synaptic inhibition and enhancement (Kakegawa and Yuzaki, 2005), and the blockade of their interaction rapidly reduces the amplitude of excitatory postsynaptic potentials (EPSCs) mediated by AMPARs (Lin and Sheng, 1998). The essential role of NSF in zebrafish embryos has also been confirmed, as NSF is involved in the transport and regulation of the GluR2 subunit, and manipulation of NSF activity affects AMPA-mediated excitatory postsynaptic currents (Patten and Ali, 2009). In summary, NSF is indispensable in the molecular regulation of AMPARs, encompassing synapse stability, calcium permeability, CARP, and LTD/LTP modulation.

##### 2.2.3.2 DOPAR

*N*-ethylmaleimide-sensitive factor binds to the C-terminal of dopamine receptors D1 and D5, potentially participating in the recycling process of D1-like receptors, regulating their internalization and re-expression (Heydorn et al., 2004). The interaction between D1 receptors and NSF is crucial for receptor membrane localization. Disruption of this interaction reduces the membrane localization of D1 receptors and inhibits cAMP accumulation (Chen and Liu, 2010). Activation of D2 receptors leads to a direct interaction between D2 receptors and NSF, disrupting the interaction between NSF and the GluR2 subunit of AMPARs. It further decreases the expression of AMPARs on the cell membrane, enhances the interaction between GluR2 and the PI3K p85 subunit, and activates PI3K. Disrupting the interaction between D2 and NSF results in the loss of D2 receptors’ ability to attenuate AMPA-mediated neurotoxicity (Zou et al., 2005).

##### 2.2.3.3 GABAR

*N*-ethylmaleimide-sensitive factor regulates the cell surface expression of gamma-aminobutyric acid (GABA) A receptors in hippocampal neurons by interacting with residues 395–415 of the GABA A receptor *β* subunit. This interaction impacts the availability of AMPARs in synapses, synaptic plasticity, and memory formation (Goto et al., 2005). Additionally, NSF binds to GABA B Receptor 2 (GBR2), with the binding region being a 27-amino acid sequence at the C-terminal of GBR2 (Pep-27). NSF prebinds to GBR2 to activate the receptor, promoting the phosphorylation of the GBR2 receptor by protein kinase C (PKC), resulting in agonist-induced receptor desensitization (Pontier et al., 2006).

Through its ATPase activity, NSF regulates the assembly and disassembly of SNARE complexes, a common mechanism affecting the trafficking of AMPAR, GABAR, and DOPAR. This unifying role of NSF underscores its importance in maintaining synaptic plasticity and neurotransmission across different types of synapses. The dysfunction of NSF-mediated trafficking of these receptors is associated with several neurological disorders. For instance, altered NSF-AMPAR interactions can contribute to cognitive deficits in Alzheimer’s disease, while impaired NSF-GABAR modulation is linked to epilepsy. Understanding the shared and specific mechanisms of NSF’s interaction with these post-synaptic receptors can provide insights into therapeutic targets for these conditions.

### 2.3 Regulation of NSF activity through post-translational modification

Research indicates that the activity of NSF in cells is regulated through post-translational modifications, which allow for temporal and spatial control of membrane fusion (Huynh et al., 2004). Various cell types utilize specific post-translational modifications of NSF to achieve localized regulation of membrane fusion. Moreover, NSF can undergo reversible inactivation through phosphorylation and *S*-nitrosylation (Morgan and Burgoyne, 2004).

#### 2.3.1 Phosphorylation

The activity of NSF is tightly regulated through phosphorylation and dephosphorylation processes (Huynh et al., 2004; Liu et al., 2006). Previous studies have demonstrated that depolarization-induced NSF phosphorylation occurs in brain synaptosomes, suggesting its occurrence in neurons (Matveeva et al., 2001). Pctaire1, a member of the cyclin-dependent kinase (CDK) family, is phosphorylated and controlled by CDK5/p35 in both neuronal and non-neuronal cells. Within the NSF-D2 region, Pctaire1 phosphorylates NSF at Ser569. This phosphorylation event affects the oligomerization state of NSF: replacing Ser569 with alanine (S569A) eliminates phosphorylation and stabilizes NSF oligomers, while the S569E mutation leads to oligomerization defects (Liu et al., 2006). Tyrosine 83, located between the NA and NB subdomains of the N domain, undergoes tyrosine phosphorylation, which may affect interactions and disrupt the interface between substructures, subsequently influencing *α*-SNAP binding ability (Huynh et al., 2004; Zhao et al., 2007). Tyrosine kinases Fes and Fer phosphorylate Tyrosine 83 of NSF, while the tyrosine phosphatase PTP-MEG2 specifically dephosphorylates this residue. PTP-MEG2 functions as a positive regulator, facilitating normal NSF function by regulating the dynamic cycling of vesicle fusion through tyrosine phosphorylation and dephosphorylation at Tyr83, which is crucial for maintaining cellular function. *In vitro*, the tyrosine kinase Fes exerts dual effects on NSF phosphorylation. First, it increases the intrinsic ATPase activity of NSF. Second, it reduces NSF’s affinity for *α*-SNAP. These effects lead to the accumulation of non-functional NSF, which in turn inhibits membrane fusion (Huynh et al., 2004). PKC negatively affects NSF activity by phosphorylating it at Ser237, impacting its binding to SNAP-SNARE complexes and potentially restricting movement within the NSF functional domain, thereby influencing conformational changes in Sensor 2 associated with ATP hydrolysis (Matveeva et al., 2001). PKCε specifically regulates NSF and promotes GABA A receptor transport. Activation of the PKCε isoform leads to decreased cell surface expression of GABA A receptors and weakened GABA A currents. PKCε phosphorylates NSF at Ser460 and Thr461, enhancing NSF ATPase activity, which is critical for GABA A receptor downregulation. Treatment with a PKC activator reduces GABA-stimulated currents, while treatment with an NSF inhibitor increases the cell surface expression of GABA A receptors (Chou et al., 2010). Targeting PKCε or NSF may be a potential therapeutic strategy for restoring synaptic inhibition associated with functional impairments in GABA A receptors in neurological and psychiatric disorders.

#### 2.3.2 *S*-nitrosylation

*S*-nitrosylation is a post-translational modification that impacts the interaction of the protein NSF with the SNARE complex. Specific mutations in the cysteine residues of NSF, particularly Cys21 and Cys91, result in a decrease in its ATPase activity and impair its interaction with the SNARE complex. Furthermore, a mutation at Cys264, which is located in the ATP-binding region of NSF, impairs nucleotide binding and consequently renders the protein inactive (Matsushita et al., 2003). In endothelial cells and platelets, the *S*-nitrosylation of NSF has been observed to inhibit platelet granule secretion. However, the addition of NSF can counteract this inhibition and restore the standard functionality of secretion. In particular, *S*-nitrosylation at the Cys91 site of NSF, induced by nitric oxide (NO), enhances its binding to the AMPAR GluR2 subunit, thereby regulating receptor surface expression. The affinity of this S-nitrosylation-mediated binding between NSF and GluA2 does not depend on ATPase activity. These findings suggest that the *S*-nitrosylation of NSF may play a physiological regulatory role in NO-induced synaptic plasticity. However, it is essential to note that there are multiple potential targets for cell nitrosylation (Huang et al., 2005). Thioredoxin acts as a denitrosylase for NSF, denitrosylating it and thereby promoting exocytosis (Ito et al., 2011).

## 3 Role of NSF in synaptic function

The NSF gene is highly expressed in the central nervous system, especially in the hippocampus. Its expression begins before synaptic formation in post-mitotic neurons after embryonic division (Püschel et al., 1994). NSF primarily exists as hexameric complexes in the cytoplasm and is a crucial ATPase involved in essential biological processes within the cell. These processes encompass intracellular vesicle transport, neurotransmitter release, and synaptic plasticity. By disassembling the SNARE complex in an ATP-dependent manner, NSF facilitates the exocytosis and endocytosis of SVs. This ensures efficient neurotransmitter release and the maintenance of synaptic function. Additionally, NSF regulates the transport and expression of various neurotransmitter receptors, directly influencing synaptic plasticity. Overall, as a key ATPase, NSF plays a central role in multiple physiological functions within the nervous system.

### 3.1 Membrane fusion and secretion

*N*-ethylmaleimide-sensitive factor is a vital protein involved in intracellular membrane fusion processes in eukaryotes (Block et al., 1988; Malhotra et al., 1988). It interacts with SNARE proteins to facilitate the fusion of intracellular vesicles with the cell membrane, allowing for the transport of substances within and between cells. NSF is actively recruited to the membrane through its binding with adapter proteins SNAPs (Wilson et al., 1992). During membrane fusion, SNARE proteins assemble into α-helical trans-SNARE complexes that bring the membranes into proximity, thereby facilitating the merging of the lipid bilayers (Sudhof and Rizo, 2011). Recent findings demonstrate that an increase in SNARE complex assembly not only augments the rate of neurotransmitter release through single pores but also enables the release of larger cargos, suggesting a direct correlation between SNARE complex number and fusion pore efficiency (Bao et al., 2018). NSF, in synergy with proteins like Munc18-1, Munc13-1, and complexin-1, ensures the stability of these complexes, preventing their disassembly by NSF-αSNAP and thus maintaining vesicle priming (Prinslow et al., 2019). Moreover, NSF’s ATPase activity is pivotal for the disassembly of SNARE complexes post-fusion, allowing for their recycling and the continuation of vesicular transport (Littleton et al., 2001). The recent study reveals that NSF, in conjunction with syt1, drives the formation of committed trans-SNARE complexes that form large, stable fusion pores. These pores, once opened, can only be closed through the action of the ATPase NSF, which mediates pore closure via a complex “stuttering” mechanism (Das et al., 2020). This discovery highlights the dynamic interplay between NSF’s role in fusion pore opening and its subsequent disassembly activity, which is critical for the regulated assembly and disassembly of fusion pores.

*N*-ethylmaleimide-sensitive factor also plays a crucial role in other intracellular fusion events, such as the transport of secretory vesicles (Sztul et al., 1993), platelet granule secretion (Huynh et al., 2004; Polgár and Reed, 1999), and the release of pro-inflammatory mediators in endothelial cells (Lowenstein and Tsuda, 2006). Research has explored strategies to target NSF, such as designing specific peptides and antibody interventions, to inhibit its ATPase activity and impact secretion in human platelets and endothelial cells (Matsushita et al., 2003). Additionally, NSF is involved in the release of pro-inflammatory and prothrombotic mediators in endothelial cells by functioning as a redox sensor. In endothelial cells, the activity of NSF is regulated by nitric oxide and hydrogen peroxide through S-nitrosylation and oxidation, respectively. This regulation plays a role in influencing the mechanisms of exocytosis (Lowenstein and Tsuda, 2006). In summary, NSF plays a crucial role in various intracellular membrane fusion processes, regulating synaptic transmission and other cellular fusion events.

### 3.2 Neurotransmitter release

The NSF protein plays a critical role in regulating neurotransmitter release. Within the cytoplasm, NSF facilitates the fusion between the postsynaptic membrane and the SV membrane by disassembling the SNARE complex through the process of ATP hydrolysis. This fusion enables the orderly release of neurotransmitters (Rizo, 2022; Zinsmaier and Bronk, 2001). Studies employing a temperature-sensitive mutation in Drosophila dNSF1 known as comatose (comt) have demonstrated that under temperature-sensitive conditions, comt significantly increases the number of docked vesicles at synaptic terminals. This finding suggests that the NSF protein is involved in the maturation process of docked vesicles, ensuring the sustained release capability of neurotransmitters (Kawasaki et al., 1998).

Furthermore, NSF exerts a pivotal influence on the dynamics of neurotransmitter release, meticulously tuning both the volumetric output and the velocity of release. Recent findings highlight the intricate relationship between SNARE complex assembly and fusion pore properties, showing that altering the number of SNARE complexes can modulate the release rate through individual fusion pores, potentially impacting NSF’s role in neurotransmitter release (Bao et al., 2018). Another study utilized a photosensitive NSF inhibitory peptide and demonstrated that the NSF protein regulates neurotransmitter release through two distinct reaction rate steps. The slower step involves the decomposition of *cis*-SNARE complexes and regulates the quantity of neurotransmitter release. At the same time, the rapid action within 0.22 s affects postsynaptic currents, modulating the dynamic speed of neurotransmitter release (Kuner et al., 2008).

Moreover, research indicates that the phosphorylation level of the NSF protein, regulated by PKC, directly influences its binding affinity to SNAP-SNARE complexes. Consequently, this phosphorylation inhibits NSF activity, leading to a reduction in neurotransmitter release at synaptic terminals. This discovery elucidates a direct link between specific phosphorylation events and the modulation of neurotransmission (Matveeva et al., 2001). Another molecule called neurexin (NRX), primarily localized at the presynaptic terminal, directly interacts with the NSF protein. By influencing the distribution of the NSF protein at the presynaptic terminal and its ability to disassemble the SNARE complex, NRX regulates NSF and consequently impacts neurotransmitter release (Li et al., 2015). Additionally, the NSF protein undergoes ubiquitin modification by the mitochondrial E3 ubiquitin ligase Ariadne-1 (Ari-1). In mouse models lacking Ari-1, there is a decrease in the frequency of spontaneous neurotransmitter release but an enhancement in neurotransmitter release induced by stimulation. This suggests that Ari-1, through its modification of NSF, can selectively regulate spontaneous and stimulus-induced neurotransmitter release pathways (Ramírez et al., 2021). These research findings reveal multiple vital regulatory mechanisms of the NSF protein in modulating neurotransmitter release processes.

### 3.3 Synaptic plasticity

More reports suggest that the NSF protein plays a critical role in synaptic reorganization and plasticity. The function of NSF is essential for high-dynamic responses before SV fusion with the presynaptic membrane to release neurotransmitters (Kuner et al., 2008; Li et al., 2019). Synaptic plasticity describes molecular and morphological changes associated with learning and memory, representing an experience-dependent adaptation process that involves the adjustment of synaptic strength (Hanley, 2018). NMDARs are ionotropic receptors involved in synaptic transmission and regulating neural signals. They modulate the stability and efficacy of synaptic connections and impact synaptic plasticity (Diering and Huganir, 2018). The interaction between NSF and NMDAR allows NSF to regulate NMDAR activity, thereby influencing synaptic plasticity, which is crucial for cognitive functions such as learning and memory (Morrell et al., 2005).

Additionally, NSF relies on binding to GluR2 subunits to achieve dynamic regulation of AMPAR, which is involved in the induction and expression of LTD and LTP, thereby modulating synaptic plasticity (Gardner et al., 2005; Lim and Isaac, 2005). The stable interaction between NSF and GluR2 is also involved in mediating LTP induced by protein kinase Mf (PKMf), as well as in sustaining contextual memory within the hippocampus (Migues et al., 2014). In the lateral amygdala (LA), the interaction between NSF and GluR2 is crucial for fear memory formation and consolidation (Joels and Lamprecht, 2010). Furthermore, the schizophrenia risk gene MIR137 influences the expression of the NSF gene, thereby affecting the SV recycling process. This disruption leads to abnormal SV distribution, reduced synaptic release efficiency, and impacts the formation of synaptic plasticity (Siegert et al., 2015). Moreover, NSF is involved in the Dysbindin-dependent synaptic plasticity regulatory pathway, playing a crucial role in synaptic plasticity (Gokhale et al., 2015).

### 3.4 Autophagy

*N*-ethylmaleimide-sensitive factor is involved in the processes of autophagosome formation and vesicle fusion. Alongside other conventional membrane transport factors such as SNAP and SNARE, NSF is responsible for the formation of autophagosomes. It facilitates the fusion of heterotypic membranes between autophagosomes and lysosomes by interacting with SNARE proteins. In yeast mutants lacking Sec18 (the yeast equivalent of NSF), autophagosomes can form but fail to fuse with vesicles, thereby preventing the completion of the autophagic process (Ishihara et al., 2001). While the dissociation of the SNARE complex is crucial for the fusion between autophagosomes and lysosomal membranes, it is relatively less critical for the formation of autophagosomes (Abada et al., 2017). However, whether the ATPase activity of NSF plays a role in the later stages of mammalian cell autophagy remains unclear (Mehrpour et al., 2010). Studies have indicated that under nutrient-deficient conditions, the activity of NSF protein is inhibited, resulting in weakened binding and fusion between autophagosomes and lysosomes, consequently decreasing the rate of autophagy (Fass et al., 2006). In Drosophila, mutations in dNSF1 lead to various neurodegenerative phenotypes, including motor dysfunction, a shortened lifespan, and progressive neurodegeneration, particularly the loss of dopaminergic neurons. The neurodegenerative phenotypes are linked to the maintenance of autophagy, which is compromised by defects in lysosomal protease transport. This connection arises because the NSF protein is instrumental in the disassembly and reorganization of the SNARE complex, processes that are essential for the continuity of autophagy. The overexpression of dNSF1 can alleviate *α*-synuclein-induced toxicity in a PD model of dopaminergic neurons, highlighting the neuroprotective role of dNSF1 in autophagy and degradation pathways (Babcock et al., 2015).

## 4 Association of NSF with neurological disease

The NSF gene exhibits the highest expression levels in the central nervous system (CNS) tissues (Püschel et al., 1994). NSF’s link to neurological diseases is of great interest because it helps us comprehend the disease processes and identify potential approaches. NSF plays a critical role in synaptic transmission, essential for standard neural signal transmission. Numerous neurological disorders, such as PD and AD, are associated with abnormal synaptic transmission. Impaired or aberrant expression of the NSF protein in these diseases leads to disruptions in synaptic transmission and neuronal damage. The crucial role of dNSF1 in neuroprotection in Drosophila is significant for understanding neurodegenerative diseases (Babcock et al., 2015). Therefore, investigating the association between the NSF protein and neurological disorders helps unravel the pathophysiological mechanisms of these diseases and provides a theoretical basis for developing novel therapeutic strategies.

### 4.1 NSF and PD

The C-terminal WD40 domain of the PD-associated gene LRRK2 can interact with various proteins involved in neuronal vesicle transport, including the NSF protein. Experimental findings suggest that overexpression of the WD40 domain of LRRK2 can affect the transport and distribution of cycling SV, potentially through interactions with proteins like NSF, resulting in the onset of PD (Piccoli et al., 2014). Further research has demonstrated that LRRK2 can phosphorylate the Thr645 residue of NSF, enhancing NSF’s ATPase activity and accelerating the disassembly of its catalytic SNARE complex. T645, situated within the D2 domain of NSF, is recognized as a critical site that facilitates NSF oligomerization upon ATP binding (Matveeva et al., 1997). LRRK2 phosphorylates NSF on T645, enhances its ATPase activity, and ultimately augments its ability to dissociate the SNARE complex. The common LRRK2 mutation G2019S enhances NSF kinase activity, potentially disrupting SV dynamics through abnormal phosphorylation of NSF and contributing to the manifestation of Parkinson’s symptoms (Belluzzi et al., 2016). Studies have discovered that the LRRK2 G2019S mutation can induce the aggregation of NSF protein in brain regions such as the striatum and hippocampus. The aggregation of NSF may exert toxicity on neurons, potentially contributing to the development of PD. Additionally, with the aging of individuals, the accumulation of NSF aggregates increases, while the proteasomal and autophagic functions in the brain gradually decline, suggesting that the combined impact may be a significant mechanism underlying neuronal cell death. In mouse models expressing the LRRK2 G2019S variant, the induction of autophagy has been found to clear NSF aggregates in the nervous system, leading to improvements in motor and cognitive impairments (Pischedda et al., 2021). While no clinically relevant drugs have been identified for LRRK2-associated PD, strategies that enhance autophagy offer promising therapeutic potential.

### 4.2 NSF and AD

Traditional proteomic studies on human AD tissues and AD mouse models have shown that the NSF protein is present in neurofibrillary tangles, a neuropathological characteristic of AD. This suggests a potential involvement of NSF in the disease’s pathogenic mechanisms (Hashimoto et al., 2019). Research indicates that reduced levels of NSF hinder the fusion of autophagosomes with mature lysosomes, thus inhibiting autophagy. Consequently, this leads to the accumulation of pathological proteins such as *β*-amyloid and hyperphosphorylated tau protein, ultimately triggering AD-like pathology (Zhu et al., 2022). Proximity labeling techniques have confirmed a direct interaction between the tau protein and NSF, which leads to a dose-dependent reduction in NSF activity. NSF contributes to the stability of AMPAR through its ATPase activity, promoting the surface expression of AMPAR. Tau protein, on the other hand, affects AMPAR by inhibiting NSF activity. As AMPAR plays a crucial role in synaptic plasticity and memory formation, the dysregulation of NSF is likely to result in early synaptic dysfunction and cognitive decline in AD. Mouse models suggest that inhibiting NSF activity leads to memory loss in mice, and this memory impairment can be rescued by the absence of tau protein (Prikas et al., 2022).

### 4.3 NSF and epilepsy

Screening for epilepsy-related genes has revealed a correlation between the NSF gene and the occurrence of spontaneous seizures (Guan et al., 2001; Yin et al., 2005; Yu et al., 2002). Researchers have identified mutations in the NSF gene as a cause of early infantile epileptic encephalopathy (DEE) (Suzuki et al., 2019). In an animal model of epilepsy induced by kainic acid, researchers observed a downregulation of NSF mRNA and protein expression in the hippocampal CA1 region following spontaneous seizures (Yin et al., 2005). NSF plays a role in regulating neurotransmitter release and influences signal transduction. Elevated levels of NSF can disrupt synaptic signal transduction, leading to network dysfunction among postsynaptic neurons, and providing a neurobiological basis for seizure onset. NSF alters the function and distribution of different receptors, thereby influencing the activity of various neurotransmitter systems, which may contribute to the molecular basis of epilepsy. Studies have observed an association between elevated levels of NSF and AMPAR in synapses with spontaneous sharp waves in temporal lobe epilepsy (Herold et al., 2018). Genetic variations in the NSF gene are associated with developmental DEE. While the molecular mechanisms underlying NSF-related DEE are not yet fully understood, ongoing research continues to shed light on this complex condition. NSF mutations can disrupt intracellular membrane fusion processes through dysregulation of protein structure, leading to neurodegeneration via the mTOR pathway. Inhibiting mTOR could be a therapeutic approach for NSF-related DEE (Hayashi et al., 2023).

### 4.4 NSF and nerve injury after cerebral ischemia

Transient cerebral ischemia can lead to increased proteins such as NSF within the PSD, suggesting that cerebral ischemia may initiate protein assembly mechanisms related to NSF within the PSD. Such changes can impact the composition of postsynaptic proteins, resulting in changes in neural transmission function and selective neuronal cell damage (Hu et al., 1998). Inhibition of NSF ATPase activity can lead to the inactivation and deposition of NSF proteins, halting intracellular membrane transport activities and causing cell death in cell cultures and Drosophila models (Lu et al., 2014). Similarly, complete inactivation of NSF ATPase may interrupt intracellular membrane transport activities, resulting in delayed neuronal death after transient cerebral ischemia. Studies indicate that the injury events induced by the inactivation of NSF represent a novel and common mechanism in cerebral ischemia–reperfusion injury. Transient cerebral ischemia leads to NSF inactivation, triggering a series of lethal releases and delayed neuronal death (Yuan et al., 2018a,b). Specifically, cerebral ischemia can result in the inactivation of NSF ATPase activity, initiating a cascade of events: extensive accumulation of Golgi fragments, transport vesicles, and late endosomes, disrupting the Golgi-endosome-lysosome pathway and releasing cathepsin B (CTSB). The release of CTSB can induce changes in mitochondrial outer membrane permeability and result in cell death, ultimately contributing to cerebral ischemia–reperfusion injury (Yuan et al., 2021). Following the release of CTSB, transient cerebral ischemia triggers delayed neuronal death. During this process, NSF ATPase becomes irreversibly trapped in aggregates of inactive proteins within ischemic neurons destined to die (Yuan et al., 2018a,b). These findings suggest that the inactivation of NSF ATPase plays a crucial role in the subsequent neuronal death following transient cerebral ischemia and stroke.

## 5 Conclusion and perspectives

This article provided a comprehensive analysis of the crucial role of the NSF protein in intracellular protein transport processes. NSF directly participates in several crucial biological processes, including the cycling of SV exocytosis-endocytosis, neurotransmitter release and transmission, as well as the formation of synaptic plasticity. By interacting with proteins such as SNARE complexes, AMPAR, and neurotransmitter receptors, NSF contributes to a deeper understanding of the molecular mechanisms underlying synaptic function. Moreover, this article elucidated the close relationship between NSF and various neurological disorders, including PD, AD, and epilepsy. The dysregulation of NSF function is likely to be involved in the pathological mechanisms of these diseases. For instance, phosphorylation of NSF by LRRK2 can lead to NSF aggregation in neurons, potentially explaining the pathogenesis of PD. Furthermore, the interaction between Tau protein and NSF can alter the function and cellular localization of NSF, thereby participating in the formation of AD-related memory impairments. Future research should focus on multiple aspects, including the study of high-resolution structures of various domains of NSF to comprehensively understand its interaction mechanisms with various proteins; investigation of specific alterations of NSF in different neurological disorders, such as differences in phosphorylation or levels of aggregation at various sites; screening for small-molecule drug modulators of NSF to evaluate their protective effects in disease models; and development of targeted modification techniques for NSF to validate its regulatory role in disease-related functions. Combining multidisciplinary research approaches will be necessary to thoroughly explore the biological functions and mechanisms associated with NSF in neurological disorders. These efforts will aid in identifying new therapeutic targets and advancing the prevention and treatment of various neurological disorders.

## Glossary

AAA+: ATPases Associated with diverse cellular Activities
AD: Alzheimer’s disease
AMPA: α-amino-3-hydroxy-5-methyl-4-isoxazolepropionic acid
AMPAR: α-amino-3-hydroxy-5-methyl-4-isoxazolepropionic acid receptor
Ari-1: Ariadne-1
ATP: adenosine triphosphate
CARP: Calcium-Permeable AMPA Receptor Plasticity
CDK: cyclin-dependent kinase
CG-MALS: composition-gradient multi-angle light scattering
CNS: central nervous system
CTSB: cathepsin B
DEE: early infantile epileptic encephalopathy
DOPAR: dopamine receptors
EPSC: Excitatory Postsynaptic Potential
GABA: gamma-aminobutyric acid
GABAR: gamma-aminobutyric acid receptor
GBR2: gamma-aminobutyric acid B Receptor 2
KD: dissociation constant
LA: lateral amygdala
LRRK2: leucine-rich repeat kinase 2
LTD: Long-Term Depression
LTP: Long-Term Potentiation
NBD: Nucleotide Binding Domain
NEM: *N*-ethylmaleimide
NMDAR: *N*-Methyl-d-aspartate receptor
NRX: neurexin
NSF: *N*-ethylmaleimide-sensitive factor
PD: Parkinson’s disease
PKC: protein kinase C
PKMf: protein kinase Mf
PSD: postsynaptic density
SNAP: soluble NSF attachment protein
SNARE: soluble *N*-ethylmaleimide-sensitive factor attachment protein receptor
SRH: Second Region of Homology
SV: synaptic vesicle
syt1: Ca^2+^ sensor synaptotagmin
t-SNARE: target soluble *N*-ethylmaleimide-sensitive factor attachment protein receptor
v-SNARE: vesicle soluble *N*-ethylmaleimide-sensitive factor attachment protein receptor
β2-AR: β2-adrenergic receptor
